# Decoding Mandarin Action Verbs from EEG Using a Dual-LSTM Network: Towards Practical Assistive Brain–Computer Interfaces

**DOI:** 10.3390/s26092749

**Published:** 2026-04-29

**Authors:** Binshuo Liu, Gengbiao Chen, Lairong Yin, Jing Liu

**Affiliations:** 1 International College of Engineering, Changsha University of Science and Technology, Changsha 410114, China; liubinshuo@stu.csust.edu.cn (B.L.); 202427050108@stu.csust.edu.cn (J.L.); 2 College of Mechanical and Vehicle Engineering, Changsha University of Science and Technology, Changsha 410114, China; chengengbiao@csust.edu.cn

**Keywords:** EEG signal processing, Mandarin verb decoding, recurrent neural networks (RNN), brain–computer interfaces (BCI), assistive technology

## Abstract

Electroencephalogram (EEG)-based brain–computer interfaces (BCIs) offer a promising pathway for restoring communication. Decoding tonal languages like Mandarin from EEG remains challenging due to homophones and complex temporal dynamics. This study investigates the decoding of six high-frequency Mandarin action verbs—Chi (eat), He (drink), Chuan (wear), Na (take), Kan (look), and Dai (put on)—from EEG signals. We designed a visual-cue-based overt speech production experiment and collected EEG data from 30 participants during visually guided verb reading aloud. A recurrent neural network framework incorporating dual Long Short-Term Memory (LSTM) layers was implemented to model the long-range temporal dependencies in EEG patterns. The proposed model was compared against a traditional Common Spatial Pattern combined with Support Vector Machine (CSP-SVM) baseline. Our LSTM-based model achieved an average classification accuracy of 69.93% ± 3.07% for the six-class task, significantly outperforming the CSP-SVM baseline (36.53% ± 3.17%). Accuracy exceeded 75% under specific training conditions, including more than 15 training repetitions and a training-data proportion of 38%. Furthermore, the model attained this performance level utilizing approximately 38% of the available trial data for training, demonstrating data efficiency. The results indicate that the LSTM architecture can effectively capture the neural signatures associated with Mandarin verb processing, providing a foundation for developing practical EEG-based assistive communication technologies. The inference latency of the trained model, quantified as the post-training per-trial testing time, was under 2 s, supporting near-real-time applications.

## 1. Introduction

Recent advancements in brain–computer interfaces (BCIs) have demonstrated the feasibility of decoding speech from electroencephalogram (EEG) signals, offering transformative potential for individuals with speech motor impairments [1,2,3,4,5,6]. Although significant progress has been achieved in decoding English phonemes from EEG data [7,8], the decoding of tonal languages such as Mandarin Chinese remains largely underexplored due to its linguistic complexity. The abundance of homophones and tonal variations in Mandarin introduces substantial ambiguity in neural encoding, resulting in prolonged cognitive processing and diminished classification accuracy [9].

The neural processing of speech involves specialized cortical regions, most notably Broca’s and Wernicke’s areas, which are responsible for speech production and comprehension, respectively. Decoding speech from EEG effectively requires capturing the complex, time-evolving neural patterns generated by these and other language-related networks. However, traditional EEG analysis methods often fail to adequately model these sustained and sequential dynamics.

Current studies on Mandarin EEG-speech mapping predominantly focus on isolated characters or syllables, yielding modest accuracy rates (e.g., 38.2% for four-command classification [10] and 70% in silent reading tasks [11]). These approaches often overlook the temporal dynamics of neural responses to continuous homophones—a critical limitation for the development of real-world assistive communication systems. In addition to classification accuracy, previous EEG speech-BCI studies have emphasized the importance of practical online performance, including online covert-speech classification and speech-imagery-based BCI systems [2,10].

Long Short-Term Memory (LSTM) networks, a specialized form of RNN, are particularly suited to this challenge. By design, LSTM units mitigate the vanishing gradient problem, enabling them to learn and retain long-range temporal dependencies. This capability is critical for disambiguating Mandarin homophones, as it allows the model to integrate neural activity spanning the entire perception and semantic processing sequence, from early sensory responses in auditory cortex to later, sustained activation in language-specific areas like Broca’s area.

Traditional methods such as Common Spatial Patterns (CSP) combined with Support Vector Machines (SVM) struggle to model the non-stationary characteristics of EEG patterns elicited by Mandarin verbs. While convolutional neural networks (CNNs) have shown promise in EEG classification tasks [12,13,14,15,16,17,18], their translation-invariant architecture limits their capacity to capture the sequential dependencies intrinsic to speech-evoked neural activity. Recurrent neural networks (RNNs), especially Long Short-Term Memory (LSTM) architectures, are particularly suited to this task due to their ability to exploit long-range temporal correlations [19,20,21]. However, their application to Mandarin EEG decoding remains nascent, and no prior work has systematically addressed the homophone effect using adaptive time-series modeling.

To address this gap, we propose a novel LSTM-enhanced RNN framework for high-accuracy decoding of six high-frequency Mandarin action verbs: Chi (eat), He (drink), Chuan (wear), Na (take), Kan (look), and Dai (put on).

## 2. Related Work

### 2.1. The Challenge of Decoding Language from Neural Signals

Decoding intelligible language from non-invasive brain recordings remains a significant challenge in neuroscience and brain–computer interface (BCI) research. The core difficulty lies in bridging the gap between low-bandwidth, noisy neural signals and the high-level, structured semantics of language. Recent pioneering work has demonstrated the feasibility of reconstructing continuous language semantics from non-invasive functional magnetic resonance imaging (fMRI) data by leveraging the contextual capabilities of modern language models [22]. This work underscores a critical paradigm: advanced computational models, particularly those from artificial intelligence, are essential for interpreting the complex neural representations of speech and language. Translating this paradigm to the more practical but noisier domain of electroencephalogram (EEG) signals, especially for complex linguistic structures like Mandarin homophones, constitutes a natural and important extension of this research direction.

### 2.2. The Foundational and Transformative Role of Large Language Models

The field of natural language processing has undergone a revolution with the advent of Large Language Models (LLMs). The development of the Transformer architecture provided the foundational mechanism of self-attention, enabling the modeling of long-range dependencies in text with unprecedented efficiency [23]. Building on this, models like GPT-3 demonstrated that scaling up language models could yield remarkable few-shot and zero-shot learning abilities, moving AI systems closer to general language understanding without task-specific fine-tuning [24]. The subsequent release of efficient, open-source foundation models like LLaMA has further accelerated innovation and accessibility in the field [25]. Collectively, these LLMs exhibit capabilities in language generation, comprehension, and in-context reasoning that were previously unattainable [26]. Their proficiency in generating coherent, context-appropriate text from prompts or sparse inputs presents a transformative opportunity for assistive technologies.

### 2.3. Toward Integrated Neuroprostheses: Combining Neural Decoders with Language Models

A compelling frontier for practical BCI systems is the direct integration of neural decoders with these powerful LLMs. The traditional BCI focus on classifying a limited set of discrete commands is insufficient for restoring natural, fluent communication. The emerging paradigm envisions a hybrid architecture where a neural decoder extracts the user’s core intent or semantic tokens from brain activity, and an LLM acts as a sophisticated “language backend” to articulate this intent into fluent, grammatical sentences. Early proof-of-concept for such integration comes from high-performance invasive neuroprostheses, where decoded speech representations are used to drive both speech synthesizers and conversational language models for avatar control, significantly enhancing the richness of the communicated output [27]. Similarly, research on speech synthesis from intracranial signals utilizing deep learning models points towards the broader trend of using AI to translate neural codes into complex, user-intended outputs [4]. The work presented in this paper aligns with and contributes to the first component of this integrated vision. By developing an efficient LSTM-based decoder for Mandarin action verbs, we address the critical sub-problem of accurately extracting key semantic elements (verbs) from EEG signals. This provides a reliable and essential input stream for a downstream LLM, which could then perform the linguistic heavy lifting of sentence formation, thereby moving closer to a practical, non-invasive thought-to-text communication system.

## 3. CSP-SVM Baseline Method

### 3.1. CSP Feature Extraction

Common Spatial Pattern (CSP) is a widely used spatial filtering technique designed to enhance discriminative features in EEG signals. It achieves this by maximizing the variance differences between two signal classes, thereby improving classification accuracy [28]. CSP transforms the original multi-channel EEG data into spatial components that emphasize variations between different conditions.

Following the standard CSP procedure, the normalized covariance matrix C for a given trial data matrix *D*(*i*) of size *N* × *T* (*N* channels, *T* time points) was computed as:

(1) $$C=\frac{D\left(i\right)D^{T}\left(i\right)}{trace\left(D\left(i\right)D^{T}\left(i\right)\right)}$$

The composite spatial filters were derived from the generalized eigenvalue decomposition of the averaged covariance matrices for each class pair. The feature vector *f_j_* for the filtered signal components *Z_j_* was then calculated as:

(2) $$f_{j}=\log\left(\frac{var\left(Z_{j}\right)}{\sum_{i=1}^{2m}var\left(Z_{i}\right)}\right)$$

**Figure 1:**
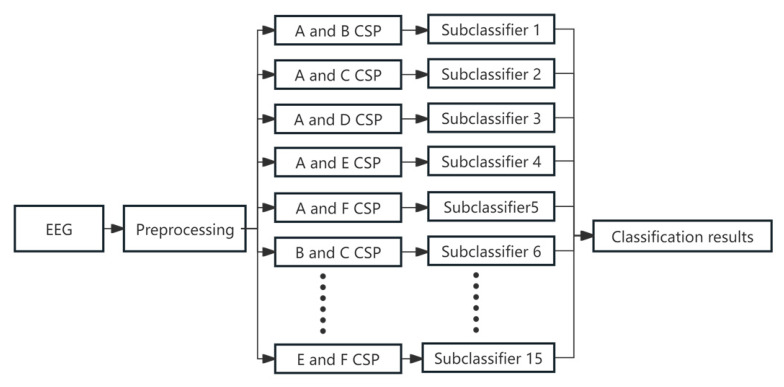
One-to-one CSP multi-class algorithm flow.

A major limitation of the standard CSP method is that it is inherently designed for binary classification tasks. To address this issue, several extensions have been developed for multi-class classification, including One-vs-One CSP [29] and One-vs-Many CSP [30]. In this study, we employed the One-vs-One CSP approach, which efficiently pairs each class against every other class without significantly increasing computational complexity. Figure 1 illustrates the multi-class EEG recognition process. Six Chinese characters—”Chi” (eat), “He” (drink), “Chuan” (wear), “Na” (take), “Kan” (look), and “Dai” (put on)—are denoted as A, B, C, D, E, and F, respectively. Using CSP, EEG data are processed to obtain 15 pairwise spatial filters. These filters are then used to extract spatial features, which are subsequently classified using Support Vector Machines (SVM).

### 3.2. SVM Classification and Recognition

Support Vector Machines (SVM) are widely used supervised learning algorithms, particularly effective for classification tasks involving limited sample sizes. SVM aims to identify an optimal hyperplane that maximizes the margin between different classes, thus enhancing classification robustness and accuracy [31].

In this study, the SVM method is applied as follows: Let *x_i_* denote the EEG feature vector for the i-th sample in the spatial domain (*x_i_*, *y_i_*) (*i* = 1, 2, …, n), *y_i_* the corresponding target class, and *n* the total number of samples. The optimal decision function *f*(*x*) is derived by solving the following optimization problem:

(3) $$f\left(x\right)=\sum_{i=1}^{n}a_{i}^{*}\cdot K\left(x_{i},x\right)+b^{*}$$

(4) $$K\left(x,x_{i}\right)=exp\left(-g{\left\|x-x_{i}\right\|}^{2}\right)$$

(5) $$w^{T}x+b=0$$

(6) $$\left\{\begin{array}{c} \underset{w,b,\xi }{\min}\left(\frac{1}{2}{\left\|w\right\|}^{2}+c\sum_{i=1}^{n}\xi_{i}\right)\\ s.t.\;\;y_{i}\left(w^{T}x_{i}+b\right)-1+\xi_{i}\geq 0\\ \xi_{i}\geq 0,\;\forall i \end{array}\right.$$

Among them, $a_{i}^{*}$ and $b^{*}$ are the optimal coefficients of classification obtained by learning; *K*(*x*,*x_i_*) is the selected radial basis kernel function, it effectively maps EEG feature vectors into a higher-dimensional space, enabling improved classification performance; *g* is the width coefficient of the applicable range of the control function; *c* is the specified constant coefficient; and $\xi_{i}$ is the relaxation factor.

## 4. RNN Recognition Based on Chinese Character Speech Mapping

**Figure 2:**
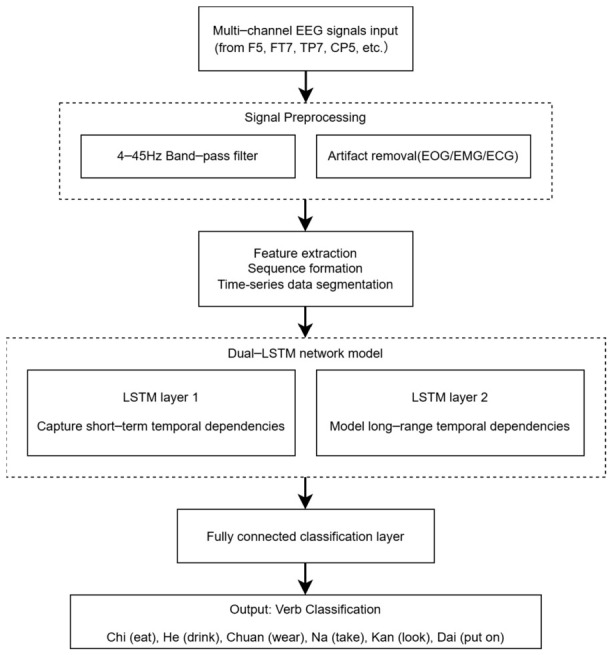
The pipeline of the proposed dual-LSTM framework for Mandarin verb decoding from EEG signals.

The overall processing pipeline of the proposed dual-LSTM network for EEG-based verb decoding is depicted in Figure 2. The framework begins with the input of multi-channel EEG signals acquired from language-related cortical areas. Subsequently, the raw signals undergo preprocessing, including band-pass filtering and artifact removal. The cleaned time-series data is then formatted into sequential segments for model input. These sequential data are fed into a dual-layer LSTM network: the first LSTM layer is designed to capture short-term temporal dependencies within the EEG patterns, while the second layer models the long-range contextual dynamics. Finally, the high-level features extracted by the LSTM layers are passed to a fully connected layer for the six-class verb classification. This structured workflow encapsulates the core methodological contribution of this study.

### 4.1. Speech Transcription

**Figure 3:**
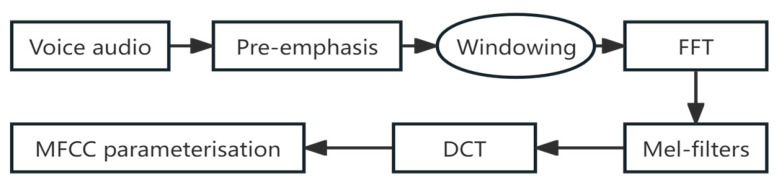
MFCC feature extraction process.

Mel Frequency Cepstral Coefficients (MFCC) [32] are widely adopted features in speech signal processing, providing a compact yet informative representation of the speech spectrum. By capturing the spectral characteristics of speech, MFCC facilitates both speech analysis and recognition. The MFCC extraction process is illustrated in Figure 3.

To guide the RNN training, reference speech features were obtained. The audio recordings of the spoken verbs were processed to extract 13-dimensional Mel-Frequency Cepstral Coefficients (MFCCs) per frame, following the standard pipeline: pre-emphasis, framing, windowing, Fast Fourier Transform (FFT) [33], Mel-filter bank application, and Discrete Cosine Transform (DCT).

### 4.2. RNN Deep Learning Model

Recurrent Neural Networks (RNNs) are specifically designed to process sequential data by maintaining a memory of prior inputs, making them particularly effective for time-series data [34]. RNNs excel at capturing temporal dependencies and dynamic patterns, which is crucial for modeling the time-varying characteristics of EEG signals in this study.

**Figure 4:**
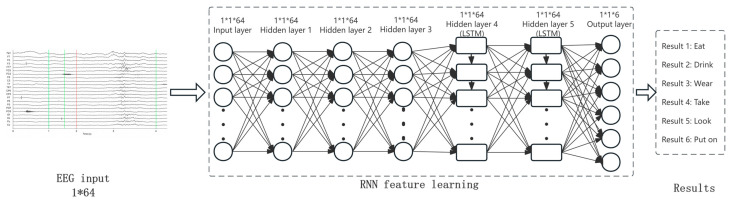
Flow chart based on RNN identification.

The RNN architecture implemented in this study, as shown in Figure 4, was developed using TensorFlow. It consists of an input layer, three fully connected hidden layers, and two Long Short-Term Memory (LSTM) layers [35]. LSTM units were incorporated to mitigate the vanishing and exploding gradient problems inherent in traditional RNNs, allowing the model to better capture long-range temporal dependencies in EEG signal data.

In this study, the network architecture was adapted from a previously established model, featuring three fully connected layers and two Long Short-Term Memory (LSTM) layers. Each hidden layer consists of multiple neurons, with the inputs subjected to linear matrix transformations. Let *X_i_* (i = 1, 2, …, 7) represent the EEG data at layer *i* of the neural network. The weight matrix between layer *i* and layer (*i* + 1) is denoted as *W_i_*_(*i*+1)_, and the bias term at layer *i* is denoted as *b_i_*. The data flow between layer *i* and layer (*i* + 1) can thus be expressed as:

(7) $$X_{i+1}=X_{i}*W_{i(i+1)}+b_{i}$$

The LSTM units, forming the fifth and sixth layers, utilized input ($f_{i}$), forget ($f_{f}$), output ($f_{o}$), and input modulation ($f_{m}$) gates to regulate information flow and mitigate the vanishing gradient problem, as described by:

(8) $$\left\{\begin{array}{c} f_{i}=sigmoid(T(X_{(i-1)j},X_{i(j-1)}))\\ f_{f}=sigmoid(T(X_{(i-1)j},X_{i(j-1)}))\\ f_{o}=sigmoid(T(X_{(i-1)j},X_{i(j-1)}))\\ f_{m}=tanh(T(X_{(i-1)j},X_{i(j-1)}))\\ c_{ij}=f_{f}\;⨀\;c_{i(j-1)}+f_{i}\;⨀f_{m}\\ X_{ij}=f_{o}\;⨀\;tanh(c_{ij}) \end{array}\right.$$

where ⨀ denotes the multiplication of the corresponding elements of two matrices; $c_{ij}$ represents the state of the *j*-th LSTM neuron in the *i*-th layer.

By controlling the information flow through these gates, LSTM helps prevent the vanishing gradient problem and improves the model’s ability to capture long-range temporal dependencies.

The following can be obtained:

(9) $$T(X_{(i-1)j},X_{i(j-1)})=X_{(i-1)j}*W_{h1}+{X_{i(j-1)}*W}_{h2}+b$$

where $W_{h1}$ and $W_{h2}$ represent the corresponding weights, respectively, and *b* represents the corresponding deviation.

Table 1 summarizes the basic configuration of the proposed RNN model, including the framework, network architecture, input settings, output classes, reference target, loss function, learning rate, and evaluation setting. This information is provided to improve the clarity and reproducibility of the proposed method.

In Figure 4, the input to the RNN is a sequence of EEG signal vectors, and the final output of the network is the predicted label among the six Mandarin action verbs. The spoken recordings were processed to extract 13-dimensional Mel-Frequency Cepstral Coefficients (MFCCs), which were used as a reference acoustic representation of the target verbs. However, the final decoding task of the proposed framework is a six-class verb classification task rather than direct MFCC-sequence prediction. During training, the network parameters were optimized by minimizing the cross-entropy loss between the predicted class probabilities and the ground-truth verb labels [36]. At each training step, a random subset of data was used to compute the loss, and the gradients were backpropagated to update the model parameters.

The cross-entropy loss function [37] quantifies the difference between the predicted MFCC sequence and the actual observed values. The function is defined as:

(10) $$Loss=-\frac{1}{n}\sum_{x}\left[y\ln a+\left(1-y\right)\ln1-a\right]$$

Among them, *x* is the sample, *a* is the predicted value, *y* is the actual observed value, and *n* is the total sample.

Minimizing the cross-entropy loss during training helps adjust the model’s weights to improve the accuracy of the predicted EEG signal features.

The choice of learning rate will have a certain impact on the training results of the RNN model. Based on the empirical formula of the gradient descent method, this study selects the best learning rate based on the criterion of maximizing the value of the loss function. It satisfies the following formula:

(11) $$\begin{array}{ll} f\left(x^{c+1}\right) & =f\left[x^{c}-\alpha_{c}\nabla f\left(x^{c}\right)\right]\\ & ={min}_{\alpha }f\left(x^{c}-\alpha \nabla f\left(x^{c}\right)\right)\\ & ={min}_{\alpha }\phi \left(\alpha \right) \end{array}$$

where $x^{c}$ and $x^{c+1}$ represent the parameters of the *c*th and (*c* + 1)th iterations, respectively, *f* is the loss function, $\alpha_{c}$ is the learning rate of the *c*th iteration, and α is the learning rate of the minimization loss function $f\left(x^{c+1}\right)$. According to Equation (12):

(12) $$\phi \prime \left(\alpha \right)=-{\left\{\nabla f\left[x^{c}-\alpha \nabla f\left(x^{c}\right)\right]\right\}}^{T}\nabla f\left(x^{c}\right)=0$$

where $\nabla f\left(x^{c}\right)$ and $\nabla f\left(x^{c+1}\right)$ denote the gradients of $x^{c}$ and $x^{c+1}$, respectively. It can be seen that the condition for minimizing the loss function $\phi \left(\alpha \right)$ is that two corresponding gradient vectors are perpendicular to each other.

## 5. Experiments

### 5.1. Experimental Subjects

The current dataset and code are publicly accessible via the OSF repository at https://doi.org/10.17605/OSF.IO/NMKE5 [38]. The study included 30 college students (15 males and 15 females), all of whom were right-handed, in good physical and mental health, with an average age of 23 ± 1.4 years. Participants were instructed to avoid alcohol and other stimulants for 24 h prior to the experiment. During the experiment, participants were seated 1 m away from the display screen in a relaxed setting, with minimal physical movement. Prior to the experiment, all participants provided informed consent.

It is noteworthy that the core experimental paradigm involved the visual presentation of Chinese characters, with participants performing visually cued reading aloud. No external auditory stimuli were presented or required to be perceived by the subjects during the EEG recording task. Therefore, parameters related to auditory perception, such as individual audiological characteristics, sound pressure level, phon, and mel scale, are not applicable to the design and interpretation of the present study, which focuses on decoding neural signals arising from visual language recognition and overt speech production. A summary of the demographic characteristics of the participant cohort is provided in Table 2.

**Figure 5:**
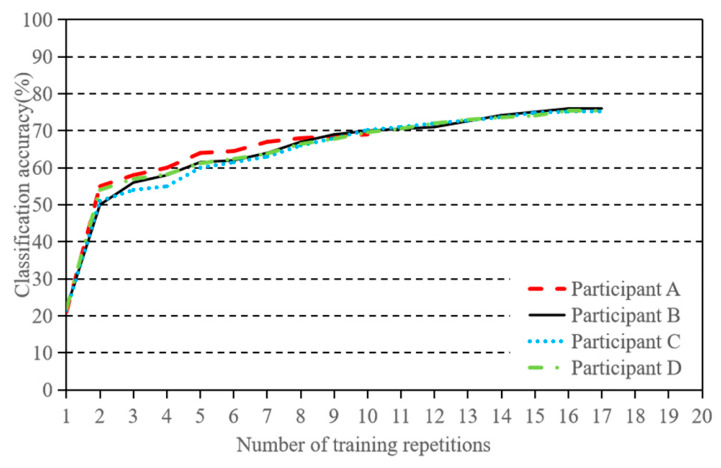
Effect of repeated training times on RNN classification accuracy.

Based on the experimental design, the complete dataset comprised 13,500 EEG trial segments (30 participants × 6 verbs × 75 trials per verb), corresponding to 450 trial segments per participant and 2250 trial segments per verb. In the data-efficiency analysis reported in Figure 5, the proportion of trial segments used for model training was gradually increased; when the training proportion reached 38%, the RNN was trained with approximately 5130 trial segments in total (about 171 trials per participant, or approximately 28–29 trials per class within each participant), while the remaining within-subject trials were reserved for offline validation/testing. The present study therefore used a subject-specific, within-dataset evaluation setting rather than a cross-subject transfer setting.

### 5.2. Data Acquisition and Processing

**Figure 6:**
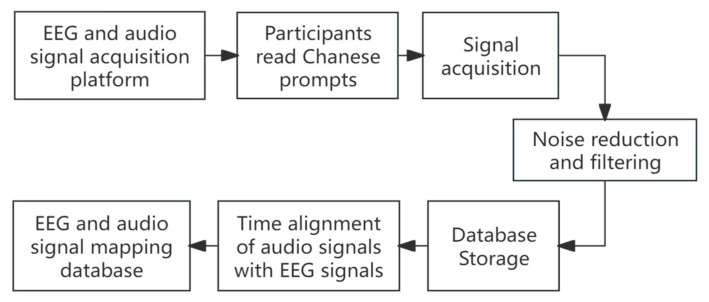
Flow chart of signal acquisition.

**Figure 7:**
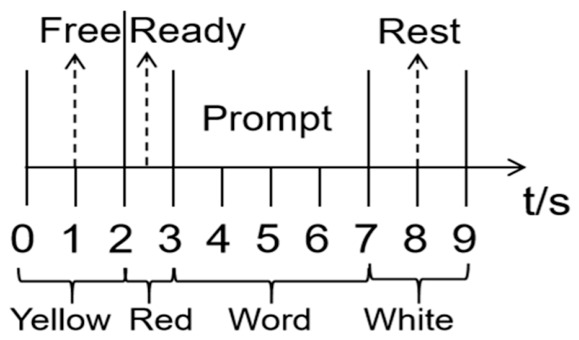
A single acquisition of the experimental sequence diagram.

This experiment uses six basic functional Chinese characters—‘Chi’, ‘He’, ‘Chuan’, ‘Na’, ‘Kan’, and ‘Dai’—as the experimental data set. Participants sat in a quiet room approximately 70 cm from a 21-inch CRT computer screen (100 Hz refresh rate). During each trial, one character briefly appears on the computer screen. The experimental timing and signal acquisition process is illustrated in Figure 6, with a detailed sequence shown in Figure 7. At the beginning of each trial, the screen displays a 2-s idle time in yellow. This is followed by a 1-s red display, signaling the participant to prepare. The target Chinese character is then displayed for 4 s while participants read it aloud. Afterward, a 2-s rest period is given, during which the screen turns white, signaling the end of the trial. Each Chinese character is shown randomly 15 times within a group of experiments. Each participant completed 5 experimental groups, with a 5-min rest period between groups. In total, 75 data points were collected per character prompt.

Due to the inherent low signal-to-noise ratio of EEG signals, data acquisition is susceptible to various sources of noise. Preprocessing steps are therefore necessary to remove artifacts that may interfere with signal quality. The primary artifacts targeted for removal in this experiment include electrooculographic (EOG) signals, electromyographic (EMG) activity, electrocardiographic (ECG) signals, and power-line interference.

The EEG data were acquired using the SynAmps System controlled by Neuroscan 4.3 software, with 64 conductive (wet) electrodes secured in an elastic cap (Electro Cap International) and placed according to the international 10/20 system [39]. The impedance of all electrodes was maintained below 5 kΩ, and the grounding electrode was positioned on the forehead to minimize 50 Hz power-line interference. The EEG signals were sampled at 250 Hz and filtered using a 4–45 Hz band-pass filter to capture relevant frequency bands (θ(4~7 Hz), α(8~15 Hz) and β(16~31 Hz)), and the filtered signals were stored digitally. Simultaneously, voice and audio signals were recorded through a dedicated 24 kHz microphone channel, using a head-mounted Shure SM35-LC cardioid condenser microphone (frequency response: 40 Hz–20 kHz), which was time-aligned with the EEG signals.

Broca’s and Wernicke’s areas are core hubs for language processing. The Broca area is primarily responsible for language production, while the Wernicke area is involved in language comprehension. In this study, brain activity from the Broca area (F5, FT7, FC5, FC3) and the Wernicke area (TP7, CP5, CP3, P5) was targeted for collection [40] to increase the likelihood of capturing relevant data while reducing the number of data channels.

## 6. Analysis of the Results

**Figure 8:**
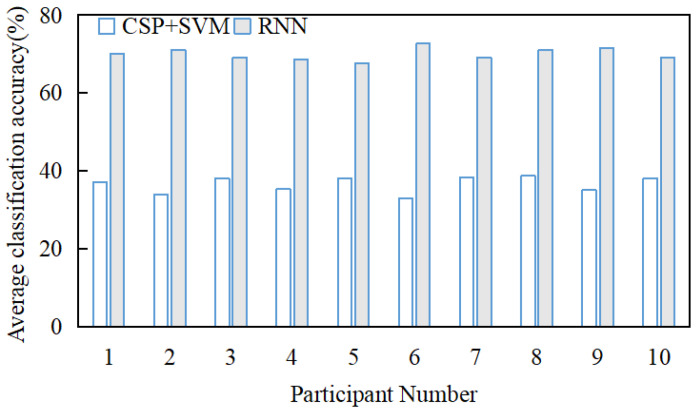
Comparison of average classification accuracy between CSP + SVM and RNN.

The performance of both methods was quantified using the average recognition accuracy throughout the experiment. Since the training and test data were drawn from the same dataset, a direct comparison of performance was possible. As shown in Figure 8, the average classification accuracy of the CSP + SVM method was 36.53 ± 3.17%, while the average classification accuracy of the RNN recognition method based on speech mapping was 69.93 ± 3.07%. These results demonstrate that the RNN-based speech mapping method achieved significantly higher accuracy compared to the CSP + SVM method.

To make the evaluation protocol explicit, overall accuracy was calculated at the trial level as the ratio of correctly classified trial segments to the total number of tested trial segments in the six-class task.

For class-wise evaluation, precision, recall, and F1-score are defined from the confusion-matrix counts in a one-versus-rest manner for each verb:

(13) $$Accuracy=\frac{\sum_{i}C_{ii}}{\sum_{i}\sum_{j}C_{ij}}$$

(14) $$Precision_{i}=\frac{TP_{i}}{TP_{i}+FP_{i}}$$

(15) $$Recall_{i}=\frac{TP_{i}}{TP_{i}+FN_{i}}$$

(16) $$F1_{i}=\frac{2\times Precision_{i}\times Recall_{i}}{Precision_{i}+Recall_{i}}$$

The recognition accuracy achieved in this study is comparable to that of other advanced methods listed in Table 3. However, the RNN-based speech mapping method proposed here is particularly suited for multi-class classification scenarios and exhibits better generalizability.

The amount of data required to achieve high-performance goals is then explored. Figure 5 illustrates the relationship between the average classification accuracy of data from four participants using the recurrent neural network (RNN) method and the number of repeated training iterations. As the number of training repetitions increases, classification accuracy improves for all participants. When the number of repetitions exceeds 15, classification accuracy surpasses 75%, reaching the threshold considered acceptable for machine translation performance.

**Figure 9:**
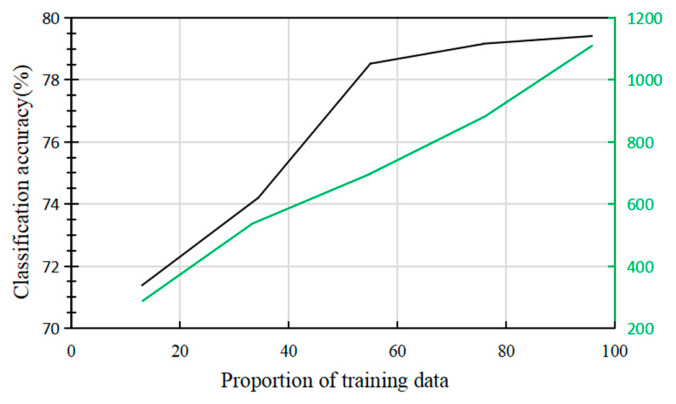
The relationship between training data proportion and RNN classification accuracy and training time.

The impact of data duplication and dataset size on model accuracy is analyzed. As shown in Figure 9, increasing the amount of training data leads to a gradual improvement in accuracy. When the proportion of training data reaches 38%, accuracy exceeds 75%. Further increases in the training data proportion result in only a marginal improvement in accuracy, suggesting that as the number of iterations increases, the method employed in this study becomes less reliant on dataset size.

Overall, deep learning models require a significant amount of time to complete tasks, which can limit their practical applicability in some scenarios. The training time for this model is shown on the right vertical axis of Figure 9. As the size of the training dataset increases linearly, the training time does not exceed 1200 s, and the model’s testing time post-training remains under 2 s. In this study, real-time performance was quantified by the post-training testing time (i.e., inference latency) required for the trained RNN to output the predicted verb label for one trial segment. This metric was evaluated separately from offline training time; therefore, the value reported in Figure 5 reflects inference efficiency after model training rather than end-to-end model optimization time. Across all tested training-data proportions, this post-training inference time remained below 2 s.

**Figure 10:**
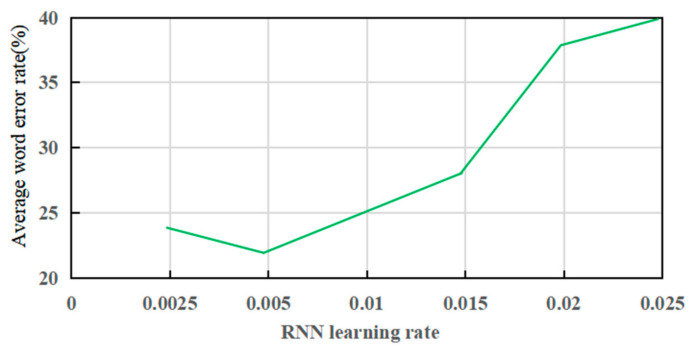
The relationship between RNN learning rate and classification accuracy.

The learning rate is a critical parameter in deep learning, determining the step size for each iteration and directly influencing whether the loss function converges to the minimum value, and how quickly it converges [47]. In this study, the average Chinese word error rate (WER) is used to quantify performance. In an ideal scenario, the WER should be 0%. Figure 10 illustrates the effect of different learning rates on the performance of the RNN model. A smaller learning rate guarantees convergence but results in slower training, consuming more time. Conversely, a larger learning rate increases the fluctuation of the loss function during training and may hinder convergence. Experimental results show that the RNN model with a learning rate of 0.005 achieves the lowest WER, yielding the best classification performance.

## 7. Discussion

In the present study, the proposed dual-LSTM framework achieved an average classification accuracy of 69.93% ± 3.07% for the six-class task, substantially outperforming the CSP-SVM baseline (36.53% ± 3.17%). Moreover, the model accuracy exceeded 75% under specific operating conditions, including more than 15 training repetitions and a training-data proportion of 38%. The achieved test compares favorably with recent EEG-based speech decoding studies involving a similar number of classes [10,11].

The superior performance of the LSTM model can be attributed to its inherent ability to model temporal dependencies. The processing of a verb unfolds over time, evoking a cascade of neural events from early visual perception to later semantic and motor-related activation. Unlike methods like CSP which extract static spatial features, or CNNs which have limited temporal receptive fields, the LSTM’s recurrent connections allow it to integrate information over the entire duration of the stimulus (4 s in our paradigm). This makes it particularly suitable for capturing the sustained and evolving neural patterns associated with verb comprehension, which is crucial for distinguishing between phonetically similar Mandarin words.

Furthermore, the model demonstrated promising data efficiency, reaching its peak performance with only 38% of the total trials used for training. This characteristic is advantageous for developing practical BCI systems, as it reduces the burden of lengthy calibration sessions on users, especially those with motor impairments. The post-training per-trial inference time of under 2 s confirms the feasibility of near-real-time application.

However, the performance gap between the proposed method and ideal accuracy highlights the ongoing challenge of EEG-based speech decoding. The non-stationary nature of EEG signals and substantial inter-subject variability in neural responses remain significant hurdles. From a neurophysiological perspective, the processing of Mandarin action verbs involves a cascade of temporally extended neural events, encompassing early perception in temporal regions to later semantic retrieval and motor-speech preparation potentially engaging Broca’s and Wernicke’s areas. Our dual-LSTM architecture is particularly suited to model this complex temporal dynamics. Unlike static spatial filters (e.g., CSP) or models with limited temporal receptive fields (e.g., CNNs), the recurrent connections and gating mechanisms of LSTM allow it to learn and integrate discriminative neural patterns over the entire stimulus duration, effectively capturing the sustained neural signatures critical for disambiguating Mandarin homophones. This alignment between the computational strength of LSTM and the neurophysiological characteristic of speech processing underpins the superior performance of our model.

## 8. Conclusions

In conclusion, the proposed dual-LSTM framework achieved an average classification accuracy of 69.93% ± 3.07% in the six-class EEG-based Mandarin verb decoding task, substantially outperforming the traditional CSP + SVM baseline. In addition, the model accuracy exceeded 75% when the number of training repetitions was greater than 15, and a comparable performance level was reached when the training-data proportion was increased to 38%. These findings indicate that the proposed method can achieve competitive performance with relatively limited training data. Additionally, the training time increased linearly with the proportion of training data, but did not exceed 1200 s. Once trained, the model’s per-trial inference time consistently remained under 2 s. By incorporating Long Short-Term Memory (LSTM) layers, the model effectively captured long-range temporal dependencies in EEG signals, addressing the challenge of homophones in Mandarin. These advancements highlight the potential of RNN-based EEG decoding for enhancing assistive communication technologies, particularly for individuals with speech motor impairments [48].

Furthermore, this work implements a dual-LSTM network architecture to model temporal dynamics for decoding Mandarin verbs from EEG, specifically designed to capture sustained and sequential neural patterns associated for disambiguating phonologically similar words. The framework demonstrates potential applicability in clinical settings where calibration data are scarce. A comprehensive processing pipeline from EEG analysis to verb classification is established, with inference times aligned to the latency requirements of practical assistive communication devices.

### 8.1. Clinical Implications

The 2-s post-training per-trial inference latency, together with the observation that the model accuracy could exceed 75% under specific training conditions, suggests the potential feasibility of the proposed framework for near-real-time assistive communication applications. However, the present findings were obtained under a subject-specific, within-dataset offline evaluation setting. Additional validation in online, closed-loop, and patient-oriented scenarios will be necessary before practical clinical deployment can be claimed.

### 8.2. Limitations and Future Work

While the results are promising, this study has several limitations that point to future research directions. First, the vocabulary was limited to six high-frequency action verbs. To move towards a practical communication device, the system must be scaled to recognize a much larger lexicon, including nouns, adjectives, and functional words, which may present different neural encoding challenges. Second, the experiment was conducted in a controlled, quiet laboratory environment. The performance of the system in noisy, real-world settings (e.g., a hospital room) must be evaluated, and robustness algorithms against environmental artifacts need to be integrated. Third, the current model is trained and tested on a per-subject basis. A critical next step is to investigate cross-subject and session-to-session generalization. Techniques such as transfer learning, domain adaptation, or the development of more invariant neural features will be essential for creating a plug-and-play system that requires minimal user-specific calibration. Finally, the use of overt visually cued overt speech production in this study differs from pure speech imagery. Future work should explicitly compare these paradigms and explore the fusion of EEG with other modalities, such as near-infrared spectroscopy, to provide a more robust decoding of speech intention.

## Figures and Tables

**Table 1:** Summary of the RNN configuration and evaluation settings.

Item	Setting Used in This Study
Framework	TensorFlow
Architecture	Input layer + three fully connected hidden layers + two LSTM layers + output layer
Input EEG channels	Eight channels from language-related regions: F5, FT7, FC5, FC3, TP7, CP5, CP3, and P5
Sampling rate	250 Hz
Trial window used for decoding	4 s task window per trial
Approximate input length	1000 time points per channel per trial (4 s × 250 Hz)
Output classes	Six Mandarin action verbs: Chi, He, Chuan, Na, Kan, and Dai
Reference acoustic representation	13-dimensional MFCCs per frame
Loss function	Cross-entropy loss
Learning rate	0.005
Evaluation setting	Subject-specific, within-dataset offline evaluation

**Table 2:** Summary of experiments and participants.

Experiment	Task	N	Sex (M/F)	Age (Mean ± SD Years)	Pictorial Stimuli	Design
Verb Decoding	recognition of visually cued action verbs	30	15/15	23 ± 1.4	Six Chinese characters: Chi (eat), He (drink), Chuan (wear), Na (take), Kan (look), and Dai (put on)	Single-factor six-level within-participants design. Each character was displayed 15 times randomly per block, with each participant completing 5 blocks.

**Table 3:** Comparison of Recognition Accuracy with Other Advanced Methods.

Authors	Methods	Classifications	Accuracy
Sun [41]	WT + SVM	2	0.65
Rashid [42]	WT + ANN	2	0.92
Major [43]	ICA + ANN	2	0.68
Vezard [44]	CSP + LDA	2	0.7159
Alaa [45]	SVM	2	0.76
Guo [11]	CSP + Fisher	4	0.7337
Ma [46]	DBN	4	0.7869

## Data Availability

To ensure full reproducibility, the complete preprocessed EEG dataset and all analysis code have been deposited in a public repository [38]. The shared data comprises the EEG signals after standard preprocessing steps, which serves as the direct input to the decoding models. This level of data sharing aligns with best practices for enabling independent verification and extension of the analytical findings presented in this work.
